# Immune surveillance and humoral immune responses in kidney transplantation – A look back at T follicular helper cells

**DOI:** 10.3389/fimmu.2023.1114842

**Published:** 2023-07-12

**Authors:** Julien Subburayalu

**Affiliations:** ^1^ Department of Internal Medicine I, University Hospital Carl Gustav Carus, Technische Universität Dresden, Dresden, Germany; ^2^ Center for Regenerative Therapies (CRTD), Technische Universität Dresden, Dresden, Germany; ^3^ Department of Medicine, University of Cambridge, Cambridge, United Kingdom

**Keywords:** HLA antigens, T follicular helper cell, transplantation immunology, solid organ transplantation, kidney transplantation, donor-specific HLA antibody, non-HLA antibody, antibody-mediated rejection

## Abstract

T follicular helper cells comprise a specialized, heterogeneous subset of immune-competent T helper cells capable of influencing B cell responses in lymphoid tissues. In physiology, for example in response to microbial challenges or vaccination, this interaction chiefly results in the production of protecting antibodies and humoral memory. In the context of kidney transplantation, however, immune surveillance provided by T follicular helper cells can take a life of its own despite matching of human leukocyte antigens and employing the latest immunosuppressive regiments. This puts kidney transplant recipients at risk of subclinical and clinical rejection episodes with a potential risk for allograft loss. In this review, the current understanding of immune surveillance provided by T follicular helper cells is briefly described in physiological responses to contrast those pathological responses observed after kidney transplantation. Sensitization of T follicular helper cells with the subsequent emergence of detectable donor-specific human leukocyte antigen antibodies, non-human leukocyte antigen antibodies their implication for kidney transplantation and lessons learnt from other transplantation “settings” with special attention to antibody-mediated rejection will be addressed.

## 1 Introduction

T follicular helper (T_FH_) cells are a specialized CD4^+^ T_H_ cell population critical for driving adaptive humoral immunity (1, 2), with B-cell lymphoma 6 (*Bcl6*) as a repressor of B-lymphocyte-induced maturation protein 1 (*Blimp-1*) driving their differentiation from CD4^+^ T helper cells (3–5). T_FH_ cells instruct germinal center (GC) formation which is pivotal to T cell-dependent antibody responses and their affinity maturation (6, 7). Inducible T cell co-stimulator (ICOS), C-X-C motif chemokine receptor (CXCR)5, and programmed cell death protein 1 (PD-1) are distinguishing markers of T_FH_ cells, which contribute to lymphoid tissue homing and B cell help (8–10). The expression of C-C motif chemokine receptor (CCR)7 instructs naïve T cell migration across high endothelial venules to locate to T cell zones. Upon activation by dendritic cells, a population of activated CD4^+^ T cells differentiates into T_FH_ cells through the up-regulation of *Bcl6* and CXCR5 with a concomitant down-regulation of CCR7 enabling them to advance toward the C-X-C motif ligand (CXCL)13-rich B cell area in lymphoid organs (11, 12). Here, Interleukin (IL)-21 and the strength of T cell antigen receptor binding determine a long-lasting antibody response through effector antibody-secreting cells (ASCs, plasma cells) and the emergence of memory B cell subsets residing in the bone marrow termed long-lived plasma cells (LLPCs) (13–18). To date, the family of T_FH_ cell subtypes comprises virus-specific T_FH_-1 cells, centrocyte-stimulating T_FH_-2 cells, IL-17- and IL-21-producing T_FH_-17 cells, and T follicular regulatory (T_FR_) cells (**Table 1**) (27). T_FH_-13 cells were recently identified which appear to be associated to IgE production to food and airborne allergens (64, 65). Synergies between these T_FH_ cell subsets with B cells provide unique and necessary cues that go beyond homeostatic B cell maturation and high-affinity antibody production (66, 67).

**Table 1:** A snapshot on T follicular cell subtypes and some aspects of their phenotypic, transcriptional, and functional characterization.

T_FH_ cell subtype	Phenotypic profile	Transcriptional profile	Function
Conventional T_FH_ cells (19–26)	CD4^+^ CD57^+^ CXCR5^+^ IL-21R^+^ IL-21^+^ ICOS^+^ OX40^+^ IL-6Rα^+^ PD-1^+^ CD69^+^ IL-2^+^ CXCL13^+^ CD40L^+^ CCR7^low^ PSGL1^low^	*Bcl-6^+^ c-Maf^+^ STAT1^+^ STAT3^+^ IRF4^+^ BATF^+^ TCF-1^+^ LEF-1^+^ TOX2^+^ ATF-3^+^ ASCL2^+^ IKZF3^+^* *Blimp-1^-^ IRF8^-^ Bach2^-^ STAT5^-^ FOXO1^-^ FOXP1^-^ KLF2^-^*	Migration to B cell zones in lymphoid tissues; antigen-specific B cell help and GC formation; B cell proliferation; immunoglobulin class-switching
T_FH_-1 cells (23, 27–36)	CD4^+^ CXCR5^+^ CXCR3^+^ ICOS^+^ PD-1^+^ CD40L^+^ CCR6^-^ IFN-γ^+^ IL-21^+^	*Bcl-6^+^ T-bet^+^ STAT1^+^ STAT3^+^ STAT4^+^*	Support of humoral and cellular immunity; antiviral support by expansion of CD8^+^ memory T cells; persistence of viral infection
T_FH_-2 cells (8, 23, 27, 37–45)	CD4^+^ CXCR5^+^ CXCR3^-^ CCR6^-^ IL-4^+^ IL-21^+^	*Bcl-6^+^ c-Maf^+^ STAT6^+^ GATA3^+^*	Class-switching to IgE; centrocyte formation, IgG4-mediated diseases; allergy; parasite/helminth infections
T_FH_-17 cells (23, 46–48)	CD4^+^ CXCR5^+^ CXCR3^-^ CCR6^+^ ICOS^+^ IL-17^+^ IL-21^+^	*Bcl-6^+^ STAT3^+^ RORγt*	ELS with spontaneous GC development; class-switching to IgG2a and IgG3; autoimmunity; wound healing
T_FR_ cells (27, 49–63)	CD4^+^ CXCR5^+^ CD25^+/-^ CD127^LOW^ FoxP3^+^ CTLA-4^+^ GITR^+^ CD28^+^ SAP^+^ ICOS^+^ Neuritin^+^	*Bcl-6^+^ ASCL2^+^ FoxP3^+^ Ezh2^+^* *STAT5^-^*	Control over follicular responses; lack of IL-4/IL-21/CD40L; differentiation from conventional T_REG_ cells or PD-1-L-dependently after immunization; modulation of extrafollicular B cell responses; control of chemokine expression from dendritic cells; imposing a brake on CD8^+^ T cell priming; surveillance of autoreactive B cell clones; suppression of autoantibodies and IgE class switching via neuritin

The table summarizes the currently reported phenotypic, transcriptional, and functional properties of T follicular helper cell subtypes. Accordingly, this table is not exhaustive.

The T_FH_-B cell interaction influences T_FH_ cells to maintain a lymph node migratory phenotype (68). Besides its auxiliary effects on B cells concerning isotype switching and the formation of GCs, IL-21 appears to support T_FH_ cells in acquiring and maintaining T_FH_ cell gene expression *in vivo* in an autocrine fashion (69–71). Under physiological conditions, the emergence of high-affinity, class-switched antibodies indicates a prosperous immune response aimed at facilitating the clearance of microbial invaders or are a means to measure successful seroconversion after vaccination (72, 73). Studies have shown that improved humoral responses were preceded by an enhanced ICOS expression on circulating T_FH_ cells (74). As such, increased ICOS^+^CXCR3^+^CXCR5^+^CD4^+^ T_FH_ cell numbers on day 7 after antigen stimulation appear to forecast a humoral response (75). Interestingly, serological response displays *Cxcr5* and *IL21* induction as early as the day of the antigenic challenge (day 0), instructs antigen-specific GC responses. A failing antigen-specific T_FH_ cell response features increased expression of *IL2* and *STAT5* (76). Accordingly, spatiotemporal positioning between T and B cell zones within GCs appears pivotal. For example, FoxP3^hi^CD4^+^ regulatory T (T_REG_) cells are predominantly confined to extrafollicular areas, whereas both T_FR_ cells and extrafollicular T_REG_ cells in vaccinated children are reduced following vaccination suggesting a released break in both peripheral T_H_ cell commitment towards T_FH_ cells (extrafollicular response) and favored cognate B cell help by T_FH_ cells within GCs (follicular response). Besides, the release of CXCL13 into the circulation reflects the ensuing GC reactivity and correlates between T_FH_ cells and Ag-specific B cells in tonsils (77), which can be considered an early surrogate biomarker for an ensuing humoral response (77, 78).

For humoral immunity, the functionality of T_FH_ cells is critical and can determine successful seroconversion. For example, during acute viral infections, fate commitment of a T_FH_ cell-to-be is acquired as early as 24 to 48 hours after infection (79). If strong interactions with antigen-presenting dendritic cells (DCs) are maintained, T_FH_ cell differentiation is facilitated permitting T_FH_ cell lineage determination over T_H_-1 cell commitment via a balanced regulation of the transcription factors *Bcl6*, T-box expressed in T cells (*T-bet*), and *Blimp-1* (2, 19, 79, 80). Also, Lee et al. have reported that the downregulation of Krüppel-like Factor 2 (*KLF2*) further promotes the inhibition of the T_FH_ cell differentiation-opposing transcription factors *Blimp1*, *T-bet*, and GATA binding protein (*GATA*)*3*, while the inhibition of *Cxcr5* transcription is withdrawn (81). IL-10 can affect the equipoise between T_H_-1 cell and T_FH_-1 cell commitment early after antigen challenge and can enhance the degree of CD4^+^ and CD8^+^ memory T cell generation and *Bcl6* expression (82).

Lately, circulating CCR7^lo^PD-1^hi^ T_FH_ cell subsets were suggested to serve as surrogate markers for an ensuing humoral response (83). Moreover, a failure to down-regulate *T-bet* in T_FH_ cells was shown to maintain an IFN-stimulated gene signature, which can propagate antigen persistence (28, 29). Hence, antigen persistence results in T_FH_-1 cell differentiation, yet may result in less meticulous B cell selection by which the origination of antigen-unspecific and self-reactive B cells and hypergammaglobulinemia is promoted (84). As a consequence, T cell exhaustion may occur (85, 86).

Lastly, the strength of T cell receptor (TCR) ligation and TCR-independent T cell activation by glycolipid recognition via CD1 contributes to determining the outcome of T_FH_ cell-driven humoral immunity (87, 88), where weak ligation maintains *T-bet* to produce B cell oligoclonality, antigen persistence, T_FH_ cell expansion, and a failing GC response.

In the context of organ transplantation, seroconversion is an undesired phenomenon since memory LLPCs residing in the bone marrow and effector ASCs within draining lymph nodes are responsible for threatening transplant longevity by imposing endothelial injury (15–18, 89). To prevent the emergence of antibodies directed against donor-specific antigenic material, donor and recipient matching for human leukocyte antigens (HLA) molecules and subjecting the recipient to an intense, life-long immunosuppression post-transplant are vital. If not adequately controlled by the immunosuppressive medication or immunodynamic interactions, the risk to develop antibody-mediated rejection (AMR) of the transplant looms. Predisposing factors for AMR include acute and chronic trauma being on hand in the transplanted setting since they disclose autoantigens, which also permit non-HLA antibodies to be patterned.

In this review, I will look back at recent and past insights into immune surveillance by T_FH_ cells following kidney transplantation, with an emphasis on clinical data. Focus will be dedicated to the involvement of T_FH_ cells in the emergence of autoantibodies against non-HLA autoantigens and *de novo* donor-specific HLA antibodies (dnDSA). Highlighting the current advances and paradigms in T_FH_ cell biology may hold the potential to stratify transplanted patients at risk for the emergence of antibodies and may open new avenues on how to treat episodes of AMR more successfully.

## 2 Kidney transplantation, organ donation after circulatory death, and the detrimental impact of ischemia reperfusion injury

Previous studies have primed our understanding of factors influencing allograft tolerance. Whilst donor age and HLA mismatches were demonstrated indicators for kidney allograft survival of deceased donors, the factor age did not apply to living donations (90). Intriguingly, the presence of dnDSA more adversely affects allograft survival in donations after circulatory death (DACD) (91). Since it is obvious that deceased donor transplants are more often subject to ischemia-reperfusion injury (IRI), organ preservation and recovery may not equally be off to a favorable start when rejection episodes, the transfer of pro-inflammatory cells, and ultimately allograft survival are concerned (92). Recently, various strategies are used in animals and humans to reduce the impact of inflammatory cells on allograft longevity. In that regard, targeting immune cell diapedesis by targeting the CD62/CD62L-axis has successfully demonstrated a reduction in the adherence of pro-inflammatory immune cells to the endothelial lining (93, 94). Similarly, a cease in T cell activation via the TIM-1-TIM-4 pathway was found to foster allograft survival after IRI in mice, which conceptually can impact on their fate commitment towards further differentiation trajectories (95).

### 2.1 Immunosuppressive drugs influence T_FH_ cells after kidney transplantation

**Figure 1:**
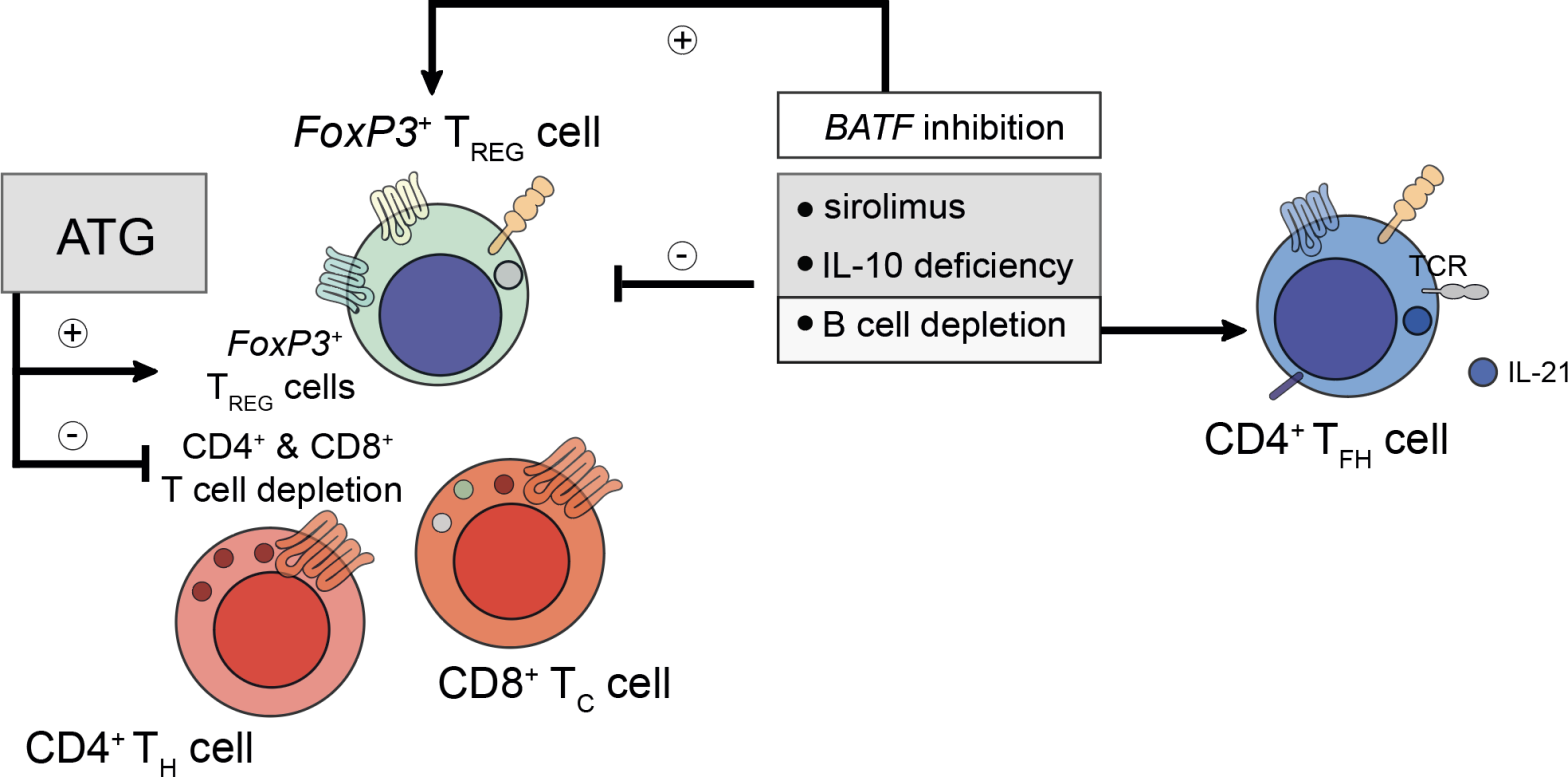
Considerations for prospective peri- and post-transplant immunosuppression. Peri- and post-transplant immunosuppressive regiments do confer differential regulatory phenotypes and need to be tailored to patient-specific demands. For example, pre-transplant administration of anti-thymocyte globulin (ATG) does deplete recipient CD4^+^ T helper (T_H_) and CD8^+^ cytotoxic T (T_C_) cells, whilst preserving the population of the recipient’s *FoxP3* ^+^ regulatory T (T_REG_) cells. T_REG_ cells are required to prevent the formation of *de novo* donor-specific human leukocyte antigen (HLA) antibodies. However, too ample T_REG_ cell-signatures do prevent the mounting of a successful humoral immune response during microbial challenge. To understand these intricacies better, future studies are needed to elucidate the mechanisms underlying the refinement and control of T_REG_, T_H_, follicular T_C_, and T follicular helper (T_FH_) cells. To date, sirolimus and interleukin (IL)-10 deficiency were found to impair T_REG_ cell activity. Likewise, B cell depletion (for example using rituximab) also disables T_REG_ cell responses. Moreover, B cell depletion restores IL-21-producing T_FH_ cell responses indirectly by alleviating the control imposed by follicular T_FR_ cells. In contrast to sirolimus and rituximab, inhibition of basic zipper ATF-like transcription factor (*BATF*) does promote T_REG_ cell responses by reducing the expression of IL-4, IL-17A, and *RORγt*.

Besides the transfer of pro-inflammatory cells, which is heightened in DACDs, the relevance of immunosuppressive drugs on T_FH_ cells after kidney transplantation and transplant longevity is being increasingly appreciated, too. Although CD4^+^ T_FH_ cell subpopulations reciprocally orchestrate their preeminent transcriptional regulation, immunosuppressive drugs can have a profound effect in governing subset skewing (96). Thymoglobulin induction therapy (e.g., antithymoglobulin/ATG) was reported to deplete effector CD4^+^ and CD8^+^ T cells, whilst preserving allograft permissive FoxP3^+^ T_FR_ cells (97, 98). This warrants further studies investigating possible therapies that draw on T_FR_ cell-mediated effects. This could potentially enable long-term drug-free allograft permissiveness (96, 99). This appears to be of particular importance since kidney transplant patients who have suffered from prior AMR were found to display higher ratios of IL-21^+^ T_FH_ cells whilst their T_FR_ cell population was decreased both within the graft and in the circulation. Besides, sirolimus was also found to reduce the T_FR_ cell population even further (100). Permissiveness may also be facilitated actively by B cells since B cell depletion or IL-10 deficiency were shown to skew the tolerogenic environment towards an increased IL-21^+^ T_FH_ cell population decreasing T_FR_ cells in follicles (**Figure 1**) (101). By extension, basic leucine zipper ATF-like transcription factor (*BATF*) inhibition, a transcription factor for T_H_-17 and T_FH_-17 cells alike, could be linked to enhanced *FoxP3* levels coinciding with a reduction in retinoic-acid-receptor-related orphan nuclear receptor gamma (*RORγt*), IL-17A, and IL-4, thus, generating tolerance after transplantation (102). Calcineurin inhibitors like cyclosporine A also impact on T_FH_ cells. Although no effect on T_FH_ subtypes concerning T_FH_-1, T_FH_-2, and T_FH_-17 cells was observed in healthy volunteers under transient cyclosporine A medication, a profound reduction in the pro-inflammatory markers IFN-γ, IL-17A, and IL-21, produced by T_FH_-1 and T_FH_-17 cells, respectively, was observed (103). This poses the question how the commonly employed permanent triple immunosuppressive therapy comprising not only of calcineurin inhibitors, but also corticosteroids and inosine-5’-monophosphate dehydrogenase (IMPDH) inhibitors, all known suppressors of T cell functionality (103–105), can lead to T_FH_ cell dysregulation culminating in alloantibody formation and potentially allograft loss.

Together, this argues for a better understanding of the effects of the lifelong post-transplant immunosuppressive regimen on the follicular and extrafollicular immune cell compartment to prevent sensitization to donor-specific HLA molecules or autologous non-HLA molecules with an eminent focus on the control of prevalent T_FH_ cell responses whilst maintaining a strong T_FR_ surveillance to induce allotolerance in kidney transplant recipients (KTxs).

### 2.2 Sequelae of persistent auto- and alloantigen stimulation of T_FH_ cells

Under physiological circumstances, the evolution of a humoral memory (LLPCs) allows high-affinity antibodies to be established within a matter of days upon re-exposure of an antigen to protect us from microbial threats (106). However, auto- and alloantigenic settings constantly expose T_FH_ cells to antigens and disturb modulating checkpoints provided by T_REG_ cells, T_FR_ cells, follicular cytotoxic T (T_FC_) cells, or even regulatory B cells. Hence, the excessive T_FH_ cell stimulation may take a life of its own. Strategies to prevent this from happening exploit cytotoxic T-lymphocyte-associated protein 4 (CTLA-4)-specific immunoglobulin or IL-21 receptor (IL-21R) antagonists to prevent alloimmune responses (107, 108).

Hence, the surveillance of immune subtype compositions may turn out as a valuable tool in identifying KTxs at risk for the formation of auto- and alloantibodies and subsequent allograft rejection.

Considering the selective influence on T_FH_ cells, studies on autoimmune diseases have shaped our understanding of persistent T_FH_ cell stimulation resulting in dysregulated responses. For example, unrestricted clonal expansion of T_FH_ cells allows the generation of ectopic lymphoid structures (ELS), which are unphysiological tertiary lymphoid follicles featuring stimulation and clonal expansion of antigen-specific B cells (109). These ELS are being established because of an antigen response yet to be cleared (110), and are formed by a regulated and well-orchestrated expression of the lymphoid chemokines C-C motif ligand (CCL)19, CCL21, and CXCL13 (111–113). Persistence of these lymphokines (with T_FH_ cells being a major source of CXCL13) (9) allows PSGL-1^LOW^CD40L^+^ T_FH_-17 cells to maintain aggregates in an ICOS-dependent manner with B cells in extrafollicular ELS, inevitably preserving an immune response with a serious risk for the formation of autoantibodies of the IgG2a/IgG2b or IgG1/IgG3 type, tissue destruction, and ultimately the development of an autoantibody-mediated pathology (114–116). Autoimmune conditions and others have been shown to be associated with ELS, which are a major compartment for ample extrafollicular T_FH_ cell accumulation (117). To date, several studies have implied T_FH_ cells especially in the context of glomerulonephritis where IL-17 drives inflammation and autoantibody-induced kidney injury which can be considered to be key determinants for autoimmune disease activity (118–121), whilst a disrupted T_FH_ cell response has been shown to reduction disease activity, respectively (122–124). It appears intuitive that especially endothelial injury and incessant inflammation with enhanced HLA class II up-regulation can expose autoantigens for T_FH_ cell sensitization (125, 126). Despite the predominance of literature, particularly from human studies, on the importance of ELS for chronic inflammation caused by infections, autoimmune diseases, cancer, or transplantation (127), the contribution of derailed T_FH_ cell activity in the induction of autoimmunity within secondary lymphoid tissues (SLOs) including the spleen, lymph nodes, and mucosal-associated lymphoid tissue (MALT) (128), may be equally important. For example, a murine study investigating the importance of T_FH_ cells in the induction of rheumatoid arthritis found T_FH_ cell frequencies to be significantly increased in the spleen and joint-draining lymph nodes following disease induction. Here, CXCR5^-/-^ mice were protected from autoimmune arthritis by abrogating T_FH_-B cell interactions within SLOs stressing its importance in the induction of autoimmune inflammation (129). In fact, not only murine models show that immune responses in SLOs may not only precede the formation of ELS and tertiary lymphoid organs (TLOs) (130), but also maintain an active role during autoimmune conditions as is observed in patients suffering from rheumatoid arthritis, who feature follicular hyperplasia and active GC responses in SLOs (131–133).

Together, a skewed T_FH_ cell response featuring lack of T_FH_-17 cell control can lead to the establishment of ELS enhancing the likelihood of T_FH_-17-B cell aggregates within these extrafollicular spaces for alloantibodies and alloreactive LLPCs to occur. Understanding how the disrupted T_FH_ cell subset equipoise can be restored may hold therapeutic potential to circumvent the need for immunosuppressive therapies with its inherent adverse effects and risk for infections (134).

### 2.3 Donor-specific HLA antibodies and T_FH_ cells: the feared couple to answer for antibody-mediated rejection

Although more and more details are being understood regarding characteristics of HLA epitopes to perform appropriate epitope matching in clinical settings, there remains much to be learned. To date, HLA-matching in kidney transplantation is only performed for HLA-A, -B, -C, -Bw4, -Bw6, -DR, -DR51/52/53, -DQA1, - DQB1, and -DPB1 antigens (135). Nevertheless, mismatches are mostly inevitable and enhance the risk of dnDSA formation (136). However, it appears that not every epitope is equally immunogenic. Aubert et al. have found that low-level donor-specific HLA antibodies remain controversial in terms of predicting the risk of graft failure (136, 137). Indeed, studies have proposed dnDSA directed at HLA-DQ to be a risk factor for late allograft failure that feature histological alterations in accordance with chronic AMR (138–140).

Although dnDSA appear to form at relatively low frequencies (~15%) and often only several years after transplantation, they commonly facilitate detrimental consequences (141, 142). Independent risk factors were reported to be HLA-DR mismatches and non-adherence to immunosuppressive therapy. Upon emergence, dnDSA could progress and show antibody-mediated graft injury without impaired graft function (142).

Evidence suggesting AMR to be a consequence of the presence of dnDSA effectively challenged the dogma of calcineurin inhibitor toxicity and chronic allograft nephropathy (141). In fact, capillaritis was found to forecast allograft dysfunction culminating in AMR (143–145). Not much later, Lefaucheur et al. identified four distinct patterns of kidney rejection with capillaritis in association with AMR illustrating the poorest outcomes (146). Not all dnDSA could be paired with ensuing AMR (147, 148). In that regard, dnDSA properties would matter with respect to MFI, complement-binding capacity, and IgG subclass composition (147, 149). Conversely, the clinical phenotype of AMR cannot be linked with the presence of circulating dnDSA in all cases challenging the criteria for diagnosis. In that regard, evidence comprised histological acute tissue injury, current antibody interaction with endothelium as in C4d deposition, and serological evidence of dnDSA detection or non-HLA donor-specific antibodies (150, 151). The identification of intragraft dnDSA has been proposed to enable clinicians to correctly diagnose AMR, which would not meet these criteria (151, 152). However, if serologically detectable, dnDSA presence has been attributed to a poor graft outcome imposed by AMR (139, 149, 153–155).

A clear link between AMR and T_FH_ cells, particularly regarding IL-21 production, has been established (156). KTxs suffering from chronic allograft rejection were found to display distinctive increases in circulating T_FH_ cells with impaired controllability given a reduced PD-1 expression (157). Accordingly, PD-1 expression fine-tunes T_FH_ cell responses by suppressing follicular T cell recruitment, confining T_FH_ cell localization within GCs, and increasing the stringency of GC affinity selection via suppressed phosphoinositide 3-kinase activity upon PD-L1 ligation (158). Another study found stable circulating T_FH_ cell numbers with decreased IL-21 production. However, their ability to stimulate alloantigen-specific B cells to produce IgG was maintained (159). Stable numbers of T_FH_ cells have also been found by Chen et al., however, indicating a skewing toward IL-21-producing T_FH_-17 and T_FH_-2 cell subpopulations in AMR (100). To control this dysregulation of T_FH_ cells, Rodriguez-Barbosa et al. have found that whilst the CD40/L pathway could be used, the B and T lymphocyte attenuator (BTLA) pathway was dispensable (160). Exploiting the CD40/L axis revealed reduced clonal B cell expansion associated with curtailed GC-T_FH_ cell numbers and a blunted IL-21 secretion (161). It appears that challenging cellular receptor-ligand interactions between T_FH_-B cells may alleviate the burden of AMR, which studies estimate to be responsible for 30-50% of allografts to fail (156). Likewise, protection from AMR could be mediated by strengthening the control of T_FH_ cells and Ag-specific B cells by T_FR_ cells (96, 100, 101, 155).

**Figure 2:**
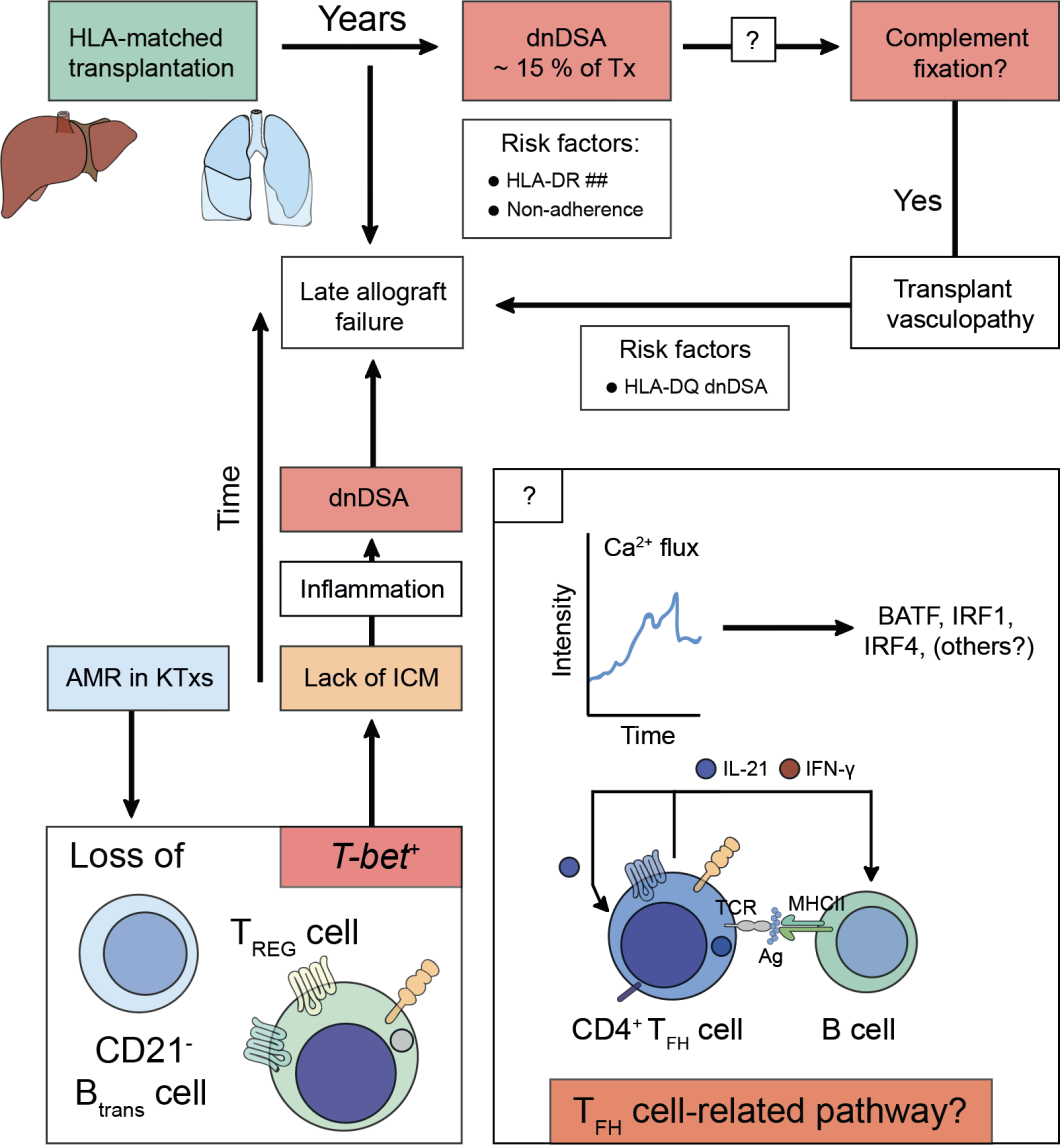
Alloimmunity following solid organ transplantation predispose for acquiring *de novo* donor-specific human leukocyte antigen antibodies and antibody-mediated allograft failure. Solid organ transplants (e.g., liver or lung) are matched for human leukocyte antigens (HLA)-A, -B, and -DR before the transplantation is undertaken. After transplantation, the constant exposure to non-HLA antigens, small antigens, or (unmatched) HLA antigens predispose for the acquisition of *de novo* Donor-specific HLA antibodies (dnDSA), which can be detected in ~ 15% of transplanted patients years after the transplantation was performed. Risk factors for the establishment of dnDSA are mismatches for HLA-DR (HLA-DR ##) or non-adherence to taking the life-long immunosuppressive treatment. The dnDSA can be classified into non-fixing or complement-fixing antibodies. Complement fixation is associated with a pro-inflammatory response, which is the cause for transplant vasculopathy. To date, it is unclear how complement-fixing abilities of dnDSA are determined (? in box), however, HLA-DQ dnDSA were found to be a risk factor. It is speculated that T follicular helper (T_FH_) cell-related pathways either on the level of fate commitment upon acquisition of antigen-specificity via T cell receptor engagement or during T_FH_ cell – B cell interactions would imprint on complement-fixing abilities of dnDSA. Irrespective of whether dnDSA can be detected in a patient, which often only attach to the transplanted vascular tissue structures of the allograft and thus evade detection by conventional blood taking methods, transplant vasculopathy drives (sub)clinical rejection episodes, which confer the risk to acquire late allograft failure. During antibody-mediated rejection (AMR) *T-bet* expression is lost from transitional B cells (CD21^-^ B_trans_ cell) and follicular regulatory T (T_FR_) cells. *T-bet* confers instructional cues for PD-1 acquisition, thus lack of immune checkpoint modulation (ICM) can be deduced that preserves an interferon-stimulated gene signature (ISGS) upon T_FH_ cell – B cell encounters. Perpetuation of ISGS maintains inflammation and fosters the establishment of dnDSA if immune suppressive measurements are not engaged.

Therefore, T_FH_ cells are not only capable of inducing dnDSA formation but can also further augment preexisting DSA levels following alloantigen recall (162). Conversely, T_FR_ cells curtail T_FH_ cell-directed B cell help by preventing dnDSA formation. Whilst lack of T_FH_ cells control drives severe AMR, T_FR_ cells are less involved in this process (162). These observations appear to translate to the human setting. Here, KTxs with immunogenic tolerance towards their allograft have disrupted T_FH_ cell functionality characterized by lack of IL-21 production, although in humans T_FR_ cells control AMR (100, 163). In a similar notion, IL-21^+^ T_FH_ cells and activated B cell responses (ASCs, CD86^+^CD38^+^) together with serum IL-21 levels were proposed as biomarkers for AMR in KTxs (164, 165). A more profound understanding if certain T_FH_ cell-related pathways predisposed to the formation of complement-fixing or non-complement fixing dnDSA, would help to develop non-invasive biomarker-guided risk stratification and molecular refinement tools to prevent the emergence of dnDSA altogether. In fact, Louis et al. recently demonstrated that T_FR_ cells and transitional B cells were selectively reduced in KTxs with AMR (166). Characteristically, both populations comprising of CXCR5^+^ T_FR_ cells and CD21^-^ transitional B cells that had vanished expressed *T-bet*. Their loss coincided with enhanced inflammatory antibody responses, microvascular inflammation, and allograft failure (166). Previous studies have highlighted the importance of *T-bet* to act as a repressor of PD-1 and other inhibitory receptors such as LAG-3, CD160, and BTLA in adaptive immune cells (167), possibly identifying *T-bet* expression as a canonical immune checkpoint to drive alloreactivity in T cells (**Figure 2**) (168). However, it must be recognized that noncanonical pathways in the absence of T_FH_-B cell interactions need to be considered that may also contribute to the production of alloantibodies or certain subtypes of allospecific immunoglobulins such as IgG2c where T_FH_ cells may be dispensable (169, 170).

Subclinical rejection episodes, a sequelae of aberrant allosensitization possibly causing loss of T_FR_ cells, are considered prognostic indicators for chronic AMR (171, 172). Adjusting signaling pathways culminating in the absence of IL-21 production in T_FH_ cells prevents dnDSA formation due to lack of cognate B cell help and thus fosters allotolerance (163). Hence, defying allosensitization of T_FH_ cells, e.g., via engagement of the co-stimulatory blockade receptor CTLA-4 (100, 173), enhanced mTOR immunosuppression (174, 175), blockade of the CD40/L axis (67, 160, 161), or antagonizing antibodies against the IL-21R (108, 156, 159), may allow to further refine or counteract chronic AMR in kidney allografts. This strategy may promote longevity of kidney transplants and the quality of life in transplanted patients. Mechanisms underlying this deleterious allosensitization of naïve T cells to becoming alloreactive T_FH_ cells are conceivable. Comparing immunosuppression with tacrolimus against co-stimulation blockade of CD80 and CD86 using the CTLA-4-Ig belatacept conferred diminished seroconversion rates to influenza vaccination (176). Hence, understanding how to prevent the selection of alloreactive T_FH_ cell effector and memory clones will be an important area of future investigations. For example, a recent cohort study in patients with a positive dnDSA status showed an enhanced alloreactive T_FH_ cell pool in response to donor-specific HLA antigens. Despite continuous immunosuppression compared with healthy controls, an augmented IL-21 production and proliferative response in these T_FH_ cells upon stimulation *in vitro* was reported suggesting either incessantly activated or more easily recruited signaling domains even years after kidney transplantation (103). Further studies are needed to better define how to circumvent unwanted allograft-directed immune responses, whilst maintaining T cell-mediated antigen-specific B cell help in the context of the host’s immune protection and response to vaccination. GC responses emerge as prominent areas to be more meticulously studied. For example, KTxs were shown to lack GC responses to mRNA vaccination against severe acute respiratory syndrome coronavirus type 2 (SARS-CoV-2), a feature well characterized to be associated with neutralizing antibodies against SARS-CoV-2 (177). A prominent feature of this missing GC response in this study was a blunted T_FH_ cell signature, which is considered instrumental in orchestrating architecture and functionality of GCs (6–10). One may speculate that allograft-draining lymph nodes’ GCs may present a unique architecture in patients having developed dnDSA compared to other allograft-unassociated draining lymph nodes. Recent studies have shown that the trauma of the surgical intervention requires lymphatic vessels to heal, which in murine kidney transplantation showed lymphatic endothelial cells to abundantly release CCL21 by which stromal lymph node remodeling was fostered. Accordingly, dendritic cell enrichment was observed thus increasing the chance for successful alloreactive T cell priming. The causative role of the lymphatic system pertaining to the kidney allograft could be established in retrospective analyses in humans (178). Furthermore, the formation of TLOs within the allograft itself were observed in human kidneys undergoing chronic rejection (179, 180). Besides the formation of *de novo* lymphatic angiogenesis, the contribution of increased lymphatic flow may be another factor by which cellular trafficking, alloimmunity, and vasculopathy are being propagated, which in the context of heart transplantation found donor cell-trafficking to allograft-draining lymph nodes, increased lymphatic vessel are, and allograft infiltration of CD4^+^ and CD8^+^ T cells as well as CD68^+^ macrophages (181). In fact, draining lymph nodes and TLOs after small-bowel transplant rejection were enriched in CXCR3^+^ host T cells stimulated by donor-derived type 1 helper T cell-related chemokines (IP-10) suggesting their possible contribution also in other solid organ transplantation contexts such as the kidney (182). Since alloreactive T cells are susceptible toward inhibition by standard immunosuppressive drugs such as corticosteroids or calcineurin inhibitors, an advanced understanding of potentially derailed signaling pathways downstream of calcineurin in cases where patients are suspected of allograft rejection despite appropriate drug levels is needed (183).

Recent studies highlight the importance to better understand cytotoxic T cell responses in GCs. Some cytotoxic T cells can acquire CXCR5 expression (28), thus enabling them to enter GCs. Compared with STAT5^+^CXCR5^+^CD8^+^ T_FC_ cells, PD-1^+^CXCR5^+^CD8^+^ T_FC_ cells were found to be a biomarker for AMR. In this study, PD-1^+^CXCR5^+^CD8^+^ T cells were associated with chronic allograft dysfunction following kidney transplantation (184). Indeed, CXCR5^+^ CD8^+^ T cells with IFN-γ-producing abilities can be sampled from the peripheral blood in higher quantities in KTxs who remain DSA-free (185). Studies in a murine system of AMR using adoptive cell transfers in CCR5 KO mice equally show reduced frequencies of CXCR5^+^CD8^+^ T_FC_ cells following AMR of the kidney transplant (186), which confer cytotoxicity towards alloprimed IgG^+^ B cells. This could be a means by which incessant adaptive immune cell activation in allograft-draining lymph nodes may be held in check in transplanted patients who remain dnDSA-free.

Together, these studies implicate both T_FH_ cell and T_FR_ cell subsets as relevant entities to risk-stratify patients concerning potential dnDSA formation (89). Further studies are needed to better define lineage-commitment trajectories of T_FC_ cells to comprehend how to therapeutically intervene in cases of ongoing AMR. This may hold the potential to maintain allograft longevity even in cases of severe AMR.

### 2.4 Non-HLA antibodies: the underestimated reason for transplant morbidity caused by exuberant sensitization of T_FH_ cells?

Having discussed the relevance of T_FH_ cells and their cellular modulators for the formation of HLA-specific dnDSA and the importance of T_FH_ cell subsets for the disclosure of autoantigens and autoimmune conditions to emerge, the relevance of non-HLA antibodies remains to be outlined.

**Figure 3:**
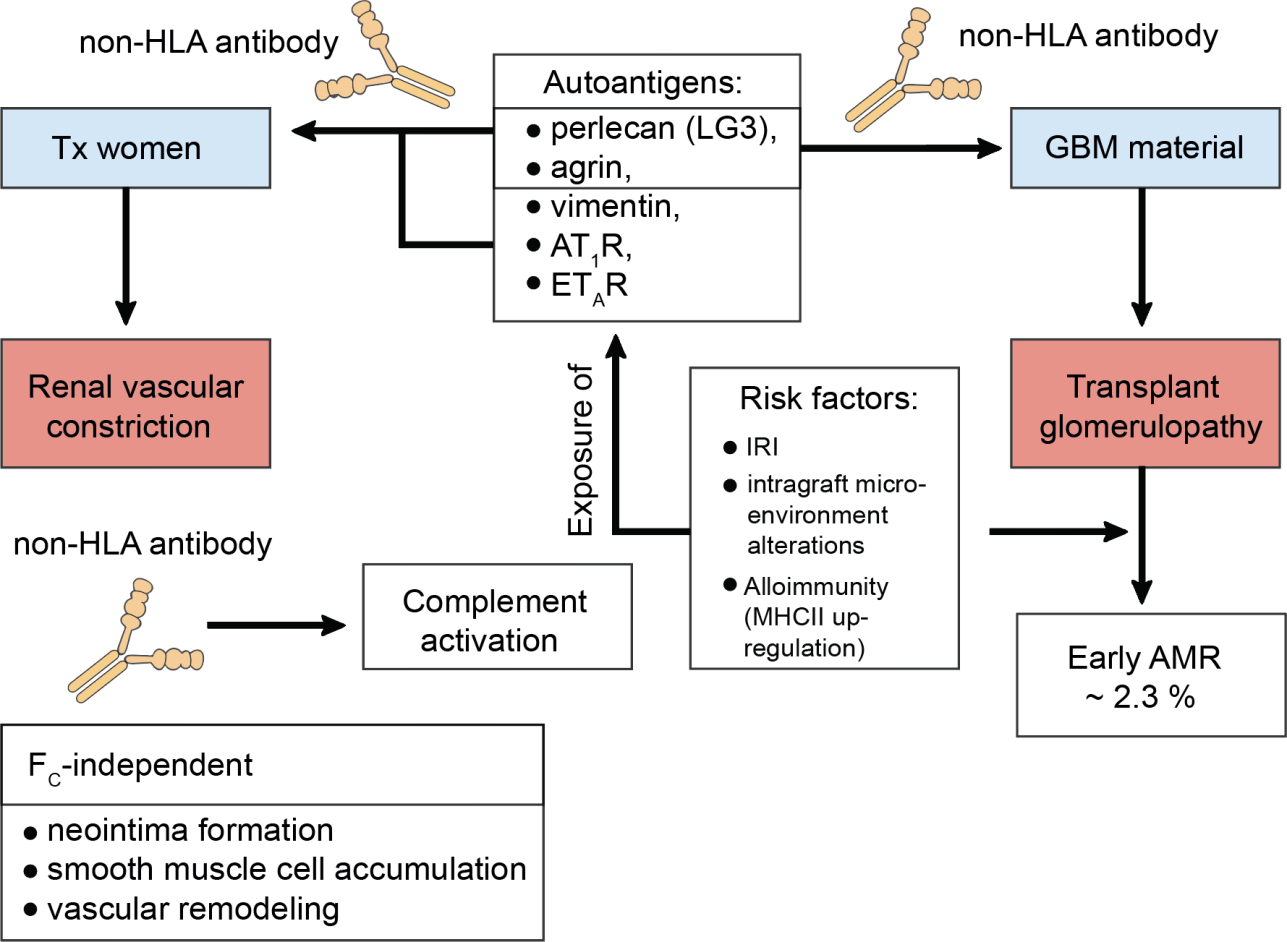
Transplant glomerulopathy in the context of non-human leukocyte antigen antibodies. Constant cellular turnover as occurs through programmed-cell death or damage to the transplanted organ predisposes for the disclosure of autoantigens. To date, the C-terminal fragment of perlecan (LG3), agrin, vimentin, the angiotensin II type 1 receptor (AT_1_R), the endothelin type A receptor (ET_A_R), k-α-1 tubulin, collagen type V, and collagen type I have been identified as serving as autoantigens with risk for progressive loss of self-tolerance. This loss of self-tolerance can result in their presentation by tissue-resident phagocytes and serological acquisition of non-human leukocyte antigen (non-HLA) antibodies without prior allosensitization. Interestingly, LG3 and agrin are composite material of the glomerular basement membrane (GBM) and can thus drive transplant glomerulopathy (TG) via renal vascular constriction, which was studies in pregnant kidney transplanted women. In this study by Hönger et al. AT_1_R was also identified to mediate TG. TG can cause early antibody-mediated rejection (AMR) and it was found that ~ 2.3% of these cases are due to non-HLA antibodies. Risk factors that mediate this process comprise ischemia-reperfusion injury (IRI), intragraft microenvironment alterations, or alloimmunity, which drives the up-regulation of major histocompatibility complex (MHC) class II (MHCII) on antigen-presenting cells, thereby upping the odds of successful cross-presentation to T helper cells. Studies have found that non-HLA antibodes can activate complement. Moreover, their pathology also entails F_C_-receptor-independent effects. For example, they can induce neointima formation, enhance the accumulation of smooth muscle cells, and promote vascular remodeling.

Just over a decade ago, Dragun et al. reported refractory vascular rejection in KTxs caused by non-HLA autoantibodies against the angiotensin II type 1 receptor (AT_1_R) (187). Similarly, transplant glomerulopathy (TG) a consequence of autoantibody formation against perlecan and agrin, both compounds of the glomerular basement membrane, was described (188). It appeared that the risk for TG was even enhanced if both dnDSA and non-HLA antibodies were coincidentally present (189). To address these descriptions, a study conducted by Amico et al. found around 2.3% of patients of their cohort, who experienced early AMR, to be due to non-HLA antibodies (190). Progressive loss of self-tolerance to the autoantigens k-α-1 tubulin, collagen type V, and the collagen I was linked to increased risk of primary graft dysfunction in lung transplantation, which in turn would augment alloimmune responses inclining towards bronchiolitis obliterans syndrome (BOS) (191, 192). The importance of activating antibodies against AT_1_R is by far the most thoroughly studied. AT_1_R and the C-terminal fragment of perlecan (LG3) were studied in pregnant transplanted women and appeared to trigger allograft rejection. The mechanism of formation did not require allosensitization and a lack of correlation suggested different mechanisms of generation (193). It was suggested that renal ischemia, alterations to the intragraft microenvironment, and alloimmunity may be some of the various factors predisposing for AT_1_R antibodies, which augment the ischemic condition by further contraction of the renal vasculature (187, 194, 195). The exposure of other cryptic antigens culminates in the emergence of non-HLA antibodies, which was described for the antigens LG3, vimentin, the endothelin type A receptor (ET_A_R), and other extracellular proteins and intermediate filaments. But also, more recently, their ability to bind to antigens present on apoptotic cells activating complement was appreciated (**Figure 3**) (195–198).

Evidence also suggests F_C_-independent effects of non-HLA antibodies like augmented neointima formation with an accumulation of smooth muscle cells, mesenchymal stem cells advocating for their regulatory function via ERK1/2 signaling and interactions with α2β1 integrins in obliterative vascular remodeling during rejection (199, 200). But also other conditions have seen the discovery of non-HLA autoantibodies including systemic sclerosis with or without associated pulmonary arterial hypertension (201, 202).

It was shown that apoptotic cells release exosome-like vesicles, which, mediated by the 20S proteasome, incite non-HLA antibody formation. This coincided with enhanced T_FH_ and GC B cells (203). The conceptual framework of apoptosis taking place constantly may allow us to explain why some non-HLA autoantibodies may be present pre-transplant. In fact, a study by Nagele et al. reported ample naturally occurring immunoglobulin (Ig) G autoantibodies, which were influenced by age, gender, and disease (204).

Together, copious amounts of studies highlight the importance of auto- and allosensitization of T_FH_ cells in generating a permissive environment to stimulate cognate Ag-specific B cells toward the production of both non-HLA autoantibodies and dnDSA. Therefore, clinicians need to carefully monitor patients to identify derailed homeostatic interactions even in the absence of measurable auto- or allosensitization (205). It is noteworthy that compartments reflecting dysbalanced interactions (ELS, allograft draining lymph nodes) are not part of the regular clinical routine suggesting that further research is needed to define appropriate biomarkers that may reflect the current state of health of these compartments. Moreover, a more fundamental understanding about how these antibodies evolve from a T_FH_ cell perspective may identify interventions potent to either decrease the intensity of the antibodies detected or even reverse their presence after all.

## 3 Lessons learnt on T_FH_ cells from other solid organ transplantation settings

Besides kidney transplantation, T_FH_ cell biology has also been studied in the context of other solid organ transplantations (**Table 2**). Although organ-specific biology considering epigenetic, molecular, cellular, and environmental effects cannot be ruled out in T cell biology, these findings can instruct experimental nephrologist to consider similar trends in kidney transplantation (215–217).

**Table 2:** A summary on the reported impact of T follicular cell subtypes in the context of solid organ transplantation settings.

Solid organ transplant	Reported T_FH_ cell subtype function
Kidney	• Increased PD-1^low^ cT_FH_ cells in chronic kidney allograft rejection (157) • Stable cT_FH_ cell numbers following transplantation (99, 158) with decreased IL-21 production (158) but enriched T_FH_-2 and T_FH_-17 cell subsets (99) • T_FH_ cell control by T_FR_ cells protects from AMR (96, 100, 101, 155) • Allotolerance inversely correlates with IL-21 production by T_FH_ cells (164, 165) • Allotolerance can be supported by CTLA-4 inhibition (100, 173), mTOR immunosuppression (174, 175), CD40/L (67, 160, 161) or IL-21R antagonism (108, 156, 159) • CXCR5^+^ T_FR_ cells and CD21^-^ transitional B cells are reduced in AMR (166) • PD-1^+^CXCR5^+^CD8^+^ T_FC_ cells correlate with AMR (184)
Heart	• mT_FH_ cells support isotype switching and alloreactivity (206) • inhibition of mT_FH_ cell trafficking prolongs allograft survival in allosensitized patients (206, 207) • IFN-γ-driven T_FH_-1 signature drives CD40-independent dnDSA formation (208), which is supported by T_FH_-17 cells (208, 209)
Lung	• CD8^+^CD44^hi^CD62L^hi^CCR7^+^ T_REG_ cells and CD4^+^CD45.2^+^FoxP3^+^ T_REG_ cells endorse allotolerance (53, 210) • T_REG_ depletion results in graft infiltration of CD4^+^Bcl6^+^CXCR5^+^PD-1^+^ T_FH_ cells, dnDSA formation, and complement activation, a phenomenon reversable by CXCL13, CD40/L, and ICOS/L inhibition (210)
Liver	• T_FH_-1- (IFN-γ) and T_FH_-17 cell signatures (IL-17) risk stratify patients for allograft rejection (211, 212) • Stable T_FH_ cell numbers before and one-month post transplantation, with IL-21^low^ T_FH_ cells post transplantation, however, unaltered Ig stimulation *ex vivo* (211, 212)
Pancreas	• Alloreactive T_FH_ cells precede insulinitis, β-cell loss, and antibody formation (213, 214)

The table summarizes the currently reported functional impact of T follicular helper cell subtypes in the context of kidney, heart, lung, liver, and pancreas transplantation. This table is not exhaustive. cT_FH_, circulating T_FH_; AMR, antibody-mediated rejection; T_FR_, T follicular regulatory cell; T_FC_, T follicular cytotoxic cell; T_REG_, regulatory T cell; mT_FH_, memory T_FH_; Ig, immunoglobulin.

**Figure 4:**
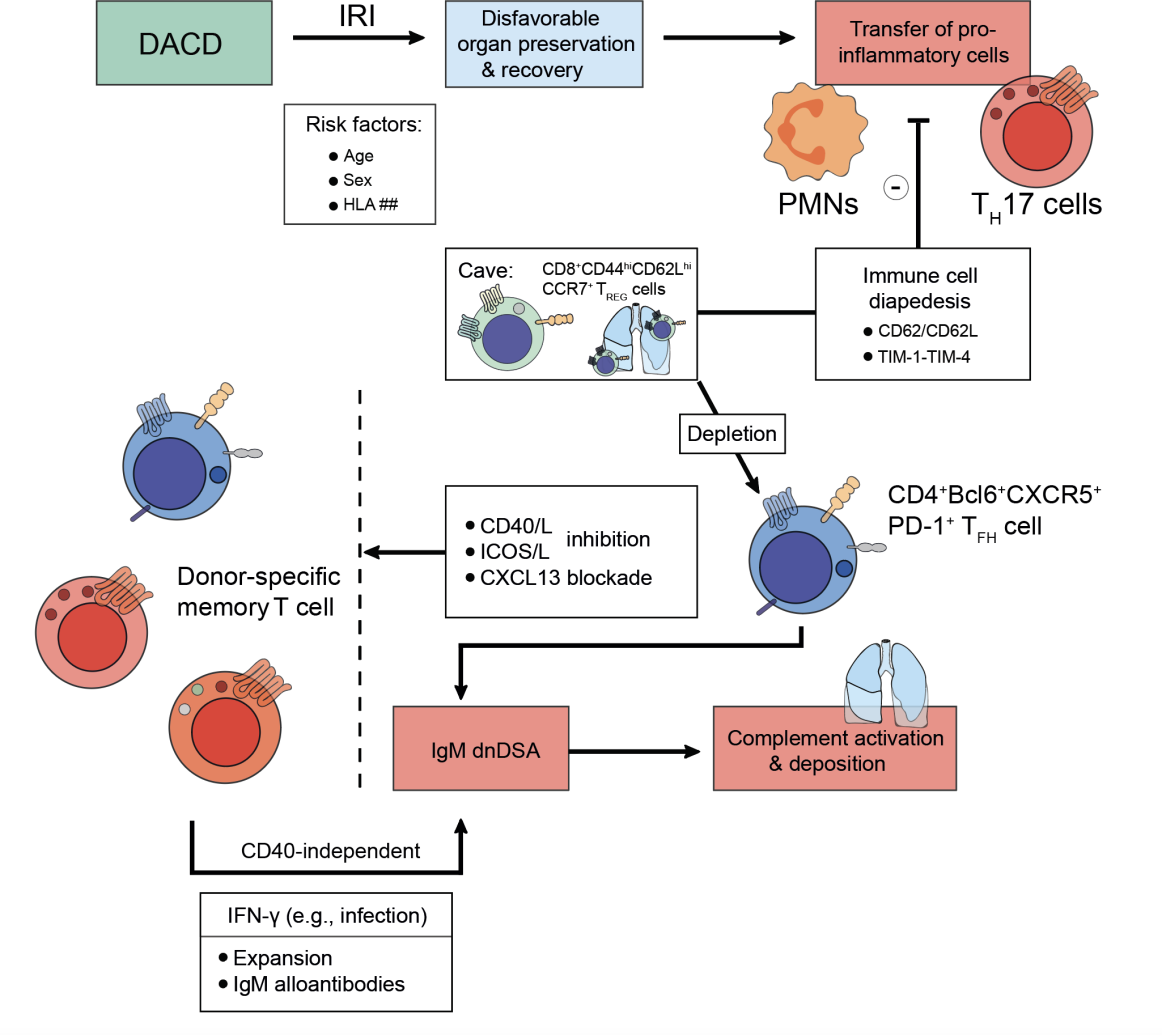
Strategies to foster transplant longevity for donations after circulatory death. Donations after circulatory death (DACD) comprise the largest fraction of organs available for transplantation. However, due to reduced circulation ischemia-reperfusion injury (IRI) has often occurred, which may be even exacerbated depending on factors including age, sex, or mismatches in the human leukocyte antigen (HLA) system. This disadvantages the length of organ preservation possible and potential chances for recovery upon transplantation. Moreover, the IRI does confer migratory effects to donor pro-inflammatory cells like polymorphonuclear neutrophils (PMNs) or IL-17A-producing T helper (T_H_17) cells, which are being transplanted jointly with the organ. However, to prevent incessant recipient pro-inflammatory immune cell recruitment towards the inflamed organ, several strategies are being explored evolving around the issue of disabling immune cell diapedesis. For example, the CD62-CD62L axis or the TIM-1-TIM-4 pathway can be targeted since CD8^+^CD44^hi^CD62L^hi^CCR7^+^ T_REG_ cells patrol the lung to endorse alloimmunity after lung transplantation. Pending successful implementation, transplant longevity may also conceptually be fostered by employing co-stimulation inhibitory approaches for the CD40/CD40L-axis, the ICOS/ICOSL-pathway, or by CXCL13 blockade to deplete donor-specific memory T cells. Following an infection that drives interferon (IFN)-γ expression, donor-specific memory T cell populations can expand and contribute to an IgM alloantibody response in a CD40-independent manner. As above, these IgM alloantibodies activate compliment and drive microvasculature inflammation that up the odds for late allograft failure.

Towards the turn of the millennium, MHC class I, mainly HLA-A, antibodies had been described to forecast the development of BOS (210, 218). Other reports reliably confirmed their detrimental impact on lung allograft longevity as they caused persistent-recurrent lung rejections and chronic allograft dysfunctions (219, 220). Moreover, dnDSA were found to occur more frequently as is the case in other solid organ transplantation settings (148). Despite their frequency, their full impact remains yet to be fully elucidated with intragraft dnDSA noticed to carry a higher risk for lung allograft loss (148). Formation of dnDSA after lung transplantation corresponds to a high risk of refractory acute cellular rejection, lymphocytic bronchiolitis (with an increased influx of lymphocytes and neutrophils in bronchoalveolar lavages) (221), and BOS (222). Lately, Krupnick et al. have described the importance of CD8^+^CD44^hi^CD62L^hi^CCR7^+^ T_REG_ cells in a mouse lung transplant model, which patrolled the lung successfully to endorse allotolerance (223). Furthermore, Li et al. have recently demonstrated that depletion of CD4^+^CD45.2^+^FoxP3^+^ T_REG_ cells induced the emergence of dnDSA resulting in AMR (155). According to the authors, T_REG_ cell depletion allowed CXCL13-mediated graft infiltration of CD4^+^Bcl6^+^CXCR5^+^PD-1^+^ T_FH_ cells and IgM dnDSA to be patterned. Ultimately, complement would deposit and destroy the airway epithelium, a process which could be reversed by CXCL13 blockade or utilization of CD40/L and ICOS/L pathway inhibition (**Figure 4**) (155). This suggests that even severe endothelial cell injury could be reversed if the right immunological setscrew was identified. These findings warrant similar research in the context of kidney transplantation. Bypassing both T_FH_ cell or T_REG_ cell influence on lung allograft longevity, a study has also successfully described B cell-targeted inhibition impeding follicle formation and eventually preventing BOS after lung transplantation (224).

In heart transplantation (HTx), the importance of alloreactive memory T_FH_ cells has been delineated previously. In that regard, memory T_FH_ cells were shown to be the barrier to long-term allograft survival as they support antibody isotype switching and alloreactive effector T_FH_ cell damage (206). Moreover, inhibiting memory CD4 T_H_ cell trafficking was assessed by Zhang et al. and effectively described to prolong HTx survival in sensitized patients with alloreactive memory T_FH_ cells, a cohort that is particularly prone to insufficient co-stimulation blockade (206, 207). By employing lymphoid sequestration, T_REG_ cell differentiation was advocated (225). More recently, Gorbacheva et al. have found that CD40-independent alloantibody responses were facilitated by IFN-γ producing T_H_ cells and may be the cause for deleterious alloantibody responses despite co-stimulation blockade (208). Especially T_H_-1 and T_H_-17 cells promoted dnDSA responses (208, 209), while also displaying CD40-independent help to alloreactive effector CD8^+^ T cells (209). Together, this suggests that follicular and extrafollicular alloreactive B cell responses need to be screened in experimental models to identify pathways by which this extrafollicular CD40-independent B cell help can be curtailed and rather host protective and allograft-permissive T cell biology can be regained.

In liver transplantation (LTx), cytokines related to T_FH_-1 and T_FH_-17 cells, IFN-γ and IL-17, have been identified to risk stratify patients regarding their risk of rejection (211, 212). More recently, T_FH_ cell dynamics were studied by Zhang et al. one day before up to one-month post-transplant. In their study, the authors have shown an unchanged T_FH_ cell frequency in the circulation, however, a reduced production of IL-21 one month after LTx was noticed. Despite the lack in IL-21 production, these T_FH_ cell could provide cognate B cell help with unaltered production of immunoglobulins in *ex vivo* cultures (213).

Lastly, pancreas transplantation utilizes similar draining lymph nodes of the paracaval and paraaortic affiliation. There are essentially three forms of pancreas transplantations (PTx) currently in practice: simultaneous pancreas-kidney (SPK), isolated pancreas (IP), and islet cell transplantation. As with other forms of organ transplantation, the deleterious impact of alloreactive T_FH_ cells was also investigated regarding tissue reactivity and alloantibody production. In a study conducted by Vendrame et al., tissue-reactive T_H_ cells were described to precede insulinitis, β-cell loss, and hyperglycemia due to C-peptide loss mediated by the emergence of autoantibodies (214). These negative consequences could be attenuated by non-specific T cell depletion (214). The challenge that alloreactive T_H_ cell pose to transplantation was delineated by another group (226). They described the serious adverse effects imposed on PTx survival by the emergence of dnDSA where, on average, 14.7% developed dnDSA, which was more frequent in IP (~ 19%) and showed an inferior outcome compared to non-donor HLA. This renders the detection of dnDSA as a strong independent predictor of pancreas allograft failure (226). Another independent study also reported in ~ 15.6% the appearance of dnDSA, predominantly class II-specific, whereas non-DSA were mostly of class I-specific nature (227). More recently, it was described that dnDSA would occur equally likely in PTx and islet cell transplantation, also being mostly class II-specific (228). Finally, more frequent, and severe rejection episodes were linked to the presence of dnDSA rather than non-DSA or antibody-negative patients (227).

## 4 Conclusions

T_FH_ cells play a crucial role in the host’s immune response and allow humoral memory to be established and maintained. However, when sensitized, particularly in a setting of persistent antigen presentation, T_FH_ cell responses are not well studied and may be surprising. Here, their responses may stretch from inabilities to mount an appropriate antibody response thus resulting in an inability to clear microbial threats or even overwhelming immune responses despite immunosuppression. The latter carries the risk of seroconversion of alloantibodies that can cause AMR. We have a lot to learn about T_FH_ cells, their interaction with B cells, T_REG_ cells, T_FR_ cells, regulatory B cells, or tissue-resident (or recruited) myeloid cells including macrophages, dendritic cells, and neutrophils. Advances in drug availability may cause a skewing of subpopulations, which may constitute an advantage or disadvantage given the respective immunological context. Taken together, progress in T_FH_ cells’ biology and unveiling their relation to CXCR5^+^CD8^+^ T_FC_ cell subsets or T_FR_ cells may hold the potential not only to risk stratify transplanted patients but moreover to regain control in cases of severe allograft injury and to reinforce allotolerance. This may bring about significant changes how we detect and treat AMR in KTxs. It is conceivable that based on an enhanced immunological understanding, AMR treatment by intensifying the immunosuppressive regimen to become a matter of the past. We should be confident that further studies can significantly improve transplant longevity, and ultimately the quality of life of transplanted patients.

## Author contributions

The author confirms being the sole contributor of this work and has approved it for publication.

## Glossary

**Table:**

Antibody-mediated rejection (AMR)	Refers to the consequences of a humoral immune response with the formation of donor-specific HLA antibodies (dnDSA), antibodies against blood group antigens or autoantigens expressed by endothelial cells upon ischemia reperfusion injury (IRI).
Bronchiolitis obliterans syndrome (BOS)	Refers to an inflamed condition resulting in an obstruction of the smallest airways that is a feared complication after lung and hematopoietic stem cell transplantation. Patients suffering from BOS experience shortness of breath, wheezing, and coughing as their lung function progressively declines.
Calcineurin inhibitors	Include drugs such as cyclosporine, tacrolimus or voclosporin. They are used as mainstay drugs after solid organ transplantation to prevent allograft injury as a consequence of the host’s overt immune activation (alloreactivity). Mechanistically, they inhibit the phosphatase calcineurin to reduce gene transcription associated with T cell activation.
Donation after circulatory death (DACD)	The most common source for organ donations, refers to the inadvertent cease in circulatory and respiratory function, by which an individual’s death (clinical death) is pronounced by physicians. At that stage, prior individual’s or family consent provided, organs and tissues can be recovered and used for organ donation.
Extrafollicular response	Refers to the growth of the lymph nodes’ paracortices or the red pulp of the spleen where naïve and memory B cells can be recruited, undergo class switching, and, to some degree, low-lever somatic hypermutation.
Follicular response	Refers to the humoral immune response mounted in germinal centers of secondary lymphoid organs (SLOs), which features clonal B cell expansion, class switching, high-affinity somatic hypermutation, B cell memory induction, and plasma cell formation.
Germinal centers	Formed structures in the B cell zone of SLOs where the follicular response is initiated.
The human leukocyte antigen (HLA) or major histocompatibility complex (MHC)	Comprises a class of type I and type II antigens which are more or less polymorphic and that encode for the cellular affiliation to one individual person.
Humoral immunity	The consequence of a successful follicular or extrafollicular response, which can be measured by antibodies produced and their seroconversion (i.e., the switch of IgM to commonly IgG molecules).
Immunosuppression	In the context of transplantation, it refers to the mainstay drugs used to prevent the rejection of the donated organ. These drugs consist of calcineurin inhibitors, corticosteroids, and others such as inosine-5’-monophosphate dehydrogenase (IMPDH) inhibitors or inhibitors of the mechanistic target of rapamycin (mTOR).
Ischemia-reperfusion injury (IRI)	Defines the worsening of cellular function and subsequent cell death because of a restored organ perfusion of previously ischemic organs (e.g., after DACD).
Islet cell transplantation	Refers to the selective transplantation of the islets of Langerhans, the parts with endocrine, i.e., insulin-producing, functionality in the definitive treatment of type I diabetes mellitus.
Isolated pancreas (IP) transplantation	Refers to the isolated transplantation of the pancreas without combined kidney transplantation in the treatment of type I diabetes mellitus.
Simultaneous pancreas-kidney (SPK) transplantation	A procedure by which both the pancreas and a kidney are simultaneously transplanted to treat kidney failure related to type I diabetes mellitus.
Transplant glomerulopathy (TG)	The histological alteration in the absence of immune depositions of the glomerular basement membrane within the Bowman's capsule of the kidney, which is most often observed in AMR.
Thymoglobulin or antithymoglobulin (ATG)	An immunosuppressive immunoglobulin used for example as a prophylactic and acute treatment of a rejection of solid organ transplants.

**Table:**

Ag	Antigen
AMR	Antibody-mediated rejection
ASC	Antibody-secreting cell
AT_1_R	Angiotensin II type 1 receptor
BAL	Bronchoalveolar lavage
BATF	Basic leucine zipper ATF-like transcription factor
BCL6	B-cell lymphoma 6
Blimp-1	B-lymphocyte-induced maturation protein 1
BOS	Bronchiolitis obliterans syndrome
BTLA	B and T lymphocyte attenuator
CCL	C-C motif ligand
CCR	C-C motif chemokine receptor
CD	Cluster of differentiation molecule
CTLA-4	Cytotoxic T-lymphocyte-associated protein 4
CXCL	C-X-C motif ligand
CXCR	C-X-C motif chemokine receptor
DACD	Donation after circulatory death
DC	Dendritic cell
dnDSA	*De novo* donor-specific HLA antibody
ELS	Ectopic lymphoid structures
ET_A_R	Endothelin type A receptor
GATA	GATA binding protein
GC	Germinal center
HLA	Human leukocyte antigen
HTx	Heart transplant
ICOS	Inducible T-cell co-stimulator
IFN	Interferon
Ig	Immunoglobulin
IL	Interleukin
IP	Isolated pancreas
IRI	Ischemia-reperfusion injury
KLF2	Krüppel-like Factor 2
KTx	Kidney transplant recipient
LCMV	Lymphocytic choriomeningitis virus
LG3	C-terminal fragment of perlecan
LLPC	Long-lived plasma cell
LTx	Liver transplant
MALT	Mucosal-associated lymphoid tissue
MFI	Mean fluorescence intensity
MHC	Major histocompatibility complex
mRNA	Messenger ribonucleic acid
PD-1	Programmed cell death protein 1
PTx	Pancreas transplant
RORγt	Retinoic-acid-receptor-related orphan nuclear receptor gamma
SARS-CoV-2	Severe acute respiratory syndrome coronavirus type 2
SLOs	Secondary lymphoid organs
SPK	Simultaneous pancreas-kidney
STAT	Signal Transducer and Activator of Transcription
T-bet	T-box expressed in T cells
TCR	T cell receptor
T_FC_	Follicular cytotoxic T
T_FH_	Conventional T follicular helper
T_FH_1	Type 1-like T_FH_
T_FH_2	Type 2-like T_FH_
T_FH_17	Type 17-like T_FH_
T_FR_	Follicular regulatory T helper
TG	Transplant glomerulopathy
T_H_	T helper
TLOs	Tertiary lymphoid organs
